# Hereditary spastic paraplegia with thin corpus callosum

**DOI:** 10.4103/0972-2327.48863

**Published:** 2009

**Authors:** Sujeet Raina, Jitender K. Mokta, Sanjiv Sharma

**Affiliations:** Department of Medicine, Indira Gandhi Medical College, Shimla, Himachal Pradesh - 171 001, India; 1 Department of Radiodiagnosis, Indira Gandhi Medical College, Shimla, Himachal Pradesh - 171 001, India

**Figure 1:**
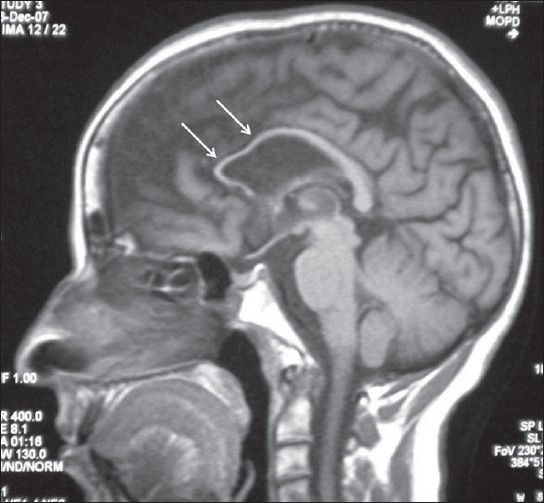
Mid-sagittal T1-weighted MR image showing thin corpus callosum mainly involving rostrum and genu (arrows) with frontoparietal lobe atrophy

**Figure 2:**
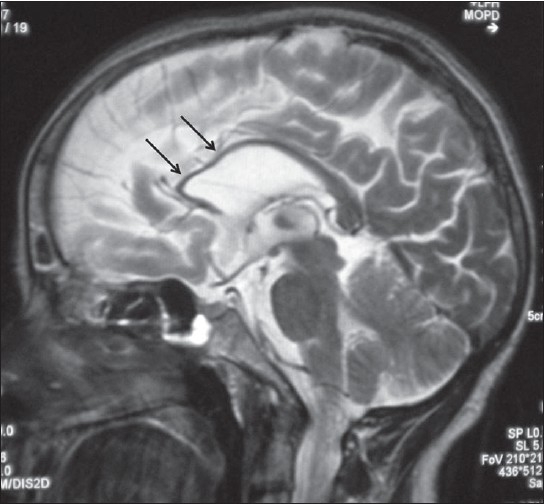
Mid-sagittal T2-weighted MR image showing thin corpus callosum mainly involving rostrum and genu (arrows) with frontoparietal lobe atrophy

**Figure 3:**
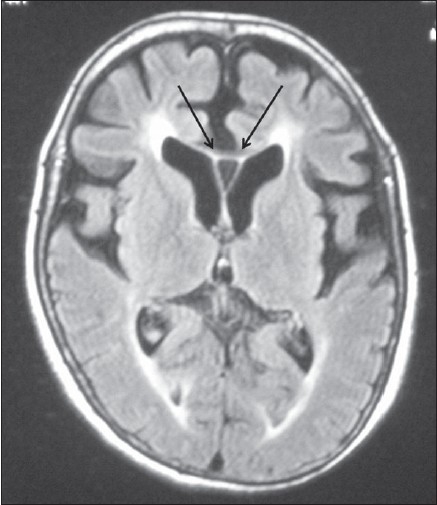
Axial FLAIR image showing white matter lesions with frontoparietal lobe atrophy with thin corpus callosum (arrows) and prominent lateral ventricle with cavum septum pellucidum

**Figure 4:**
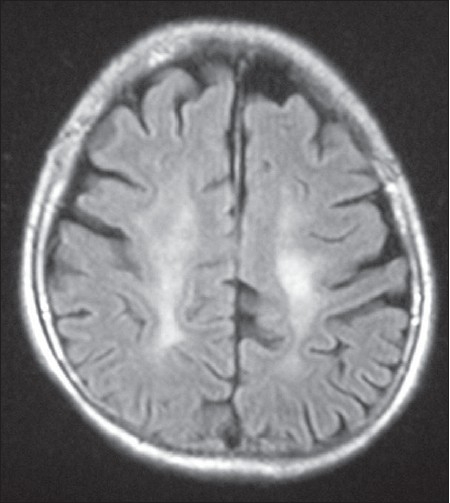
Axial FLAIR image showing white matter lesions in centrum semiovale parallel to interhemispheric fissure with frontoparietal lobe atrophy

A 42-year-old female presented with history of progressive spastic gait disturbance for the past 12 years. She had normal developmental milestones during childhood but dropped school in 12^th^ class because of low performance. She was the product of nonconsanguineous marriage and was the first of seven children in birth order. One younger female sibling has similar symptoms. No history of similar symptoms in either of the parents was present. General physical examination was normal. On central nervous system examination, patient was conscious with the evidence of impaired attention, calculation, recall, judgment, reasoning power and abstract thinking with mini mental state examination score of 23/30. Examination of cranial nerves including fundus was normal. Speech was dysarthric. She had normal muscle bulk with spasticity of lower limbs. The power in lower limbs was grade IV in all groups of muscles. All the deep tendon reflexes in both upper and lower limbs were symmetrically brisk and plantar response was bilaterally extensor. Sensory system examination was normal. She had rest tremor in upper limbs and the gait was spastic. Rest of the neurological examination was normal. On laboratory investigation, her hemogram, blood sugar, renal and liver functions, thyroid profile was found to be normal. Serum vitamin B12 was in the normal range. Cerebrospinal fluid analysis, electromyography and nerve conduction studies were normal. MRI brain and spinal cord was done, which revealed thin corpus callosum with frontoparietal cortical atrophy on the T1-weighted image [Figure 1], T2-weighted image [Figure 2] and fluid-attenuated inversion-recovery (FLAIR) MR image [Figure 3]. Symmetrical subcortical and periventricular white matter lesions were also seen on FLAIR images [Figures 3,4].

Hereditary spastic paraplegia (HSP) is a heterogeneous group of familial neurodegenerative disorders characterized by progressive lower limb spasticity. Clinically, they are classified as “pure” when spastic paraplegia exists in isolation and as complicated when other major clinical features such as mental retardation, optic atrophy, retinopathy, extra pyramidal symptoms, ataxia, deafness, cerebellar signs, muscle wasting, epilepsy, and ichthyosis are present. Genetically autosomal dominant, autosomal recessive and X-linked recessive forms of inheritance are seen with both pure and complicated forms.[1] Hereditary spastic paraplegia with thin corpus callosum classified as complicated form of spastic paraplegia was thought to be a rare neurodegenerative disorder mainly described in Japanese families with autosomal recessive transmission and the genetic locus was linked to chromosome 15q13-15[SPG11gene] which accounts for 41%–77% of reported hereditary spastic paraplegia with thin corpus callosum families and making it the most frequent cause of this disease. *SPG11* gene, also known as *KIAA1840/FLJ21439*, encodes for the protein spatacsin.[2] However, molecular genetic analyses reveal that there are several other underlying causes of this syndrome, with five other genetic loci identified (*SPG7, SPG15, SPG21, SPG32*, and HSP-TCC epilepsy).[3] Hereditary spastic paraplegia with thin corpus callosum is characterized by extremely thin corpus callosum, normal motor development, slowly progressive spastic paraparesis and dementia developing from the early second decade and other various complicated symptoms such as spastic tetraplegia, muscular atrophy, extra pyramidal symptoms, sensory impairment, cerebellar ataxia and epileptic seizures.[4]

MRI findings described are thin corpus callosum, frontoparietal atrophy and enlargement of lateral ventricles, reduced size of thalamus and symmetrical white matter lesions.[5,6] Thin corpus callosum is not specific of this syndrome and whether it represents a congenital hypoplasia or a progressive atrophy remains unknown.[5]

The diagnostic criteria of autosomal recessive HSP with thin corpus callosum are (a) autosomal recessive inheritance, (b) slowly progressive spastic paraparesis and mental impairment, (c) thinning of corpus callosum revealed by CT/MRI and (d) exclusion of other disorders by laboratory tests and MRI of spine and brain.[4] A Pubmed Medline search with key words “Hereditary spastic paraplegia, thin corpus callosum, India” revealed only one report of hereditary spastic paraplegia with thin corpus callosum from India.[7] Perhaps, cases would increase in India also after awareness of disease. Further studies are required to delineate the genetic profile of this disorder in India.
